# Effect of Hyaluronic Acid Content on Functional Properties, Antioxidant Activity, and In Vitro Digestion of Food-Grade Furcellaran Hydrogels and Emulgels

**DOI:** 10.3390/ma18245581

**Published:** 2025-12-12

**Authors:** Anna Stępień, Lesław Juszczak, Aneta Koronowicz, Aleksandra Such, Grzegorz Kowalski, Beata Synkiewicz-Musialska, Piotr Zachariasz, Ewelina Jamróz

**Affiliations:** 1 Department of Engineering and Machinery for Food Industry, Faculty of Food Technology, University of Agriculture, Balicka St. 122, PL-30-149 Kraków, Poland; grzegorz.kowalski@urk.edu.pl; 2 Department of Food Analysis and Evaluation of Food Quality, Faculty of Food Technology, University of Agriculture, Balicka St. 122, PL-30-149 Kraków, Poland; rrjuszcz@cyf-kr.edu.pl; 3 Department of Dietetics and Food Studies, Faculty of Science and Technology, Jan Długosz University in Częstochowa, Armii Krajowej 13/15, PL-42-200 Częstochowa, Poland; 4 Department of Human Nutrition and Dietetics, Faculty of Food Technology, University of Agriculture, Balicka St. 122, PL-30-149 Kraków, Poland; aneta.koronowicz@urk.edu.pl (A.K.); aleksandra.such@student.urk.edu.pl (A.S.); 5 Łukasiewicz Research Network—Institute of Microelectronics and Photonics, LTCC Technology, Zabłocie 39, 30-701 Kraków, Poland; beata.synkiewicz.musialska@imif.lukasiewicz.gov.pl (B.S.-M.); piotr.zachariasz@imif.lukasiewicz.gov.pl (P.Z.); 6 Department of Chemistry, Faculty of Food Technology, University of Agriculture, Balicka St. 122, PL-30-149 Kraków, Poland; ewelina.jamroz@urk.edu.pl; 7 Department of Packaging and Logistic Processes, Cracow University of Economics, Rakowicka 27, PL-31-510 Kraków, Poland

**Keywords:** evening primrose oil, large scale deformation, rheology, texture profile analysis, composite gels, scanning electron microscopy

## Abstract

**Highlights:**

**What are the main findings?**

- HA addition increased stability and modified swelling in gels
- HA boosted antioxidant activity before and after digestion
- Gels showed elastic-solid behavior across rheological tests

**What is the implication of the main finding?**

- HA enhances bioactive delivery potential of composite gels
- Materials suitable for hydrophilic and lipophilic compound carriers
- Systems show promise for food, cosmetic and pharma uses

**Abstract:**

Gel biocomposites, with their wide range of properties, are increasingly popular in many industries, while hyaluronic acid (HA), due to its unique water-binding mechanisms, has a high application potential in these types of materials. Furcellaran-based composite hydrogels and emulsion gels with different HA additions were produced and the effect of HA concentration on physical, color, textural, mechanical, rheological, and antioxidant properties was evaluated. A polysaccharide network was observed, which—according to Fourier transform infrared spectroscopy(FTIR) and X-ray diffraction (XRD) data—is stabilized by hydrogen bonding. Emulsion gels revealed denser structures. Small deformation tests confirmed elastic–solid type of all investigated gels. The opposite effect of HA on the swelling behavior of hydro- and emulgels was observed. Increasing hyaluronic acid content resulted in elasticity enhancement and hardness reduction. Antioxidant potential of composites significantly increased with HA concentration. The obtained materials have potential applications as plat-based delivery systems for hydrophilic and lipophilic bioactive components.

## 1. Introduction

In the area of the development of biomaterials for use in food, pharmaceuticals, and cosmetology, there is growing interest in emulgels, also known as emulsion gels, emulsion-filled gels, or gelled emulsions. These are solid-like materials composed of a three-dimensional structure made mostly of macromolecules in which the particles of the dispersed phase are immobilized [1]. In this study, hydrogels are defined as three-dimensional hydrophilic polymer networks formed exclusively in the aqueous phase, whereas emulgels refer to biphasic gel systems in which an oil-in-water emulsion is immobilized within the polysaccharide matrix. Depending on the substances employed, emulgels can exhibit diverse physicochemical, textural, or rheological properties, allowing almost unlimited design flexibility. The undoubted advantage of this type of material, in contrast to hydrogels, is its stability while containing both hydrophilic and lipophilic compounds. Hence, commercial applications of emulsion gels can include carriers of bioactive substances and controlled-release drugs, topical formulation, and fat mimetics [2,3]. There are many reports on the uses of different types of proteins [4] or carbohydrates [5] and their composites to form matrices of emulsion gels; however, research involving the employment of algal furcellaran is so far rather limited.

Furcellaran (FUR) is an anionic polysaccharide obtained from the red alga Furcellaria lumbricalis. Its structure consists of (1 → 3)-linked β-D-galactopyranose units carrying a sulfate group at the C-4 position, together with (1 → 4)-linked 3,6-anhydro-α-D-galactopyranose residues. Owing to its ability to transition from a solid (coil) state to a gel (helix) during cooling or when in the presence of ions, it is widely used as a gelling and thickening agent in food applications [6]. FUR has attracted considerable attention in biomaterial research because it is biocompatible, non-toxic, and biodegradable. The presence of multiple reactive functional groups—including carboxyl, hydroxyl, amide, and sulfate moieties—makes it a promising component for composite systems intended for bioactive compound delivery, biodegradable films, and micro- or nanocapsules [7]. Previous studies have demonstrated that the functional behavior of furcellaran can be tailored by combining it with other polysaccharides [8], proteins [9], or oils [10]. However, information is still lacking on how interactions between FUR and hyaluronic acid influence the properties of gels and emulsion-based gels.

Hyaluronic acid (HA) is a linear, anionic glycosaminoglycan built from repeating units of D-glucuronic acid and N-acetylglucosamine. Its hydrophilicity, together with the presence of hydroxyl and carboxyl groups, contributes to its excellent water solubility and strong moisture-retaining capacity. In aqueous systems, electrostatic repulsion arising from the negatively charged carboxyl groups, along with intramolecular hydrogen bonding, promotes the formation of expanded coil-like structures with a high hydrodynamic volume [11]. In the human body, HA naturally exists in the form of sodium hyaluronate and is widely distributed in synovial fluid, the extracellular matrix, connective tissues, the vitreous humor, and respiratory mucosa. Because its biodegradability, biocompatibility, non-immunogenic character, and anti-inflammatory and antibacterial activities are well established, HA is widely used in drug delivery systems, tissue engineering, and cosmetic formulations. However, its application in food-related hydrogels—and especially in emulsion-based hydrogels—remains relatively underexplored [12]. The behavior of emulsions containing hyaluronic acid has been reported for systems stabilized by various biopolymers, including casein [13], whey protein isolate [14], cellulose [15], and collagen [16].

Evening primrose oil (EPO), obtained through cold pressing or solvent extraction of Oenothera biennis seeds, is recognized for its beneficial health effects due to its unique composition and bioactive constituents. It contains high levels of omega-6 fatty acids, primarily linoleic acid (60–80%) and γ-linolenic acid (8–14%), both essential for proper physiological functioning. Studies indicate that bioactive compounds present in EPO can help alleviate chronic inflammatory conditions such as atopic dermatitis and rheumatoid arthritis. Additionally, EPO has shown therapeutic potential in managing disorders including atopic eczema, premenstrual syndrome, diabetic neuropathy, multiple sclerosis, coronary artery disease, kidney disorders, gastrointestinal problems, and endometriosis [17,18].

Previous studies have shown that the functional properties of furcellaran composite gels can be effectively modified by the appropriate addition of protein [19] or vegetable oil [20]. The unique properties of hyaluronic acid, mainly related to its hydration, make it a promising FUR-compatible component for innovative biodegradable materials with great application potential. There is no report on properties of furcellaran–hyaluronic acid gels and emulsion gels; therefore, this study focuses on determining the properties of such materials in terms of their potential food applications. It was assumed that the specific interactions between these two polysaccharides, such as molecular association, can contribute to obtaining materials with innovative properties. Knowledge of the functional characteristics of such systems can contribute to identifying and exploring areas of synergy between them. Therefore, the aim of this study was to evaluate the effect of varying the addition of hyaluronic acid on the physical, rheological, mechanical, textural properties and in vitro digestive characteristics of the furcellaran-based hydrogels and emulsion gels. This study is expected to provide insights into the mechanism of hyaluronic acid binding within hydrophilic and amphiphilic furcellaran-based gel matrices. The results of this study provide clues for the development of new composite gels and their potential use as carriers of hydrophilic and hydrophobic bioactive substances with controlled release. The full characterization of functional and technological properties presented in the study will enable material design with a wide range of applications in food technology, as well as in cosmetology, pharmacy, or biomedicine.

## 2. Materials and Methods

### 2.1. Raw Materials

To produce the hydrogel and emulgel systems, the following ingredients were used as follows: furcellaran type 7000 (Est-Agar AS, Karla Village, Estonia), cold-pressed evening primrose oil (Złota Tłocznia, Kaczory, Poland), food-grade sodium hyaluronate (also referred to as hyaluronic acid/hyaluronan) with a molecular weight of 1.27 MDa (Wisapple Biotech Co., Ltd., Beijing, China), and blue spirulina powder (Green Essence, Pyrzyce, Poland). Chemicals used for the preparation of the materials were purchased from Avantor Performance Materials Poland S.A. (Gliwice, Poland), while analytical-grade reagents applied as per the described methods were obtained from Sigma-Aldrich (St. Louis, MO, USA).

### 2.2. Hydrogel and Emulgel Preparation

Three variants of each hydrogel and emulgel based on furcellaran–hyaluronic acid were produced according to the formulation shown in Table 1. The amount of hyaluronic gel with a concentration of 1.5% [*w*/*v*] added to the formulation in percentages of 5, 10, and 15%—and addressed in further sections of this paper—corresponds to 0.075, 0.150, and 0.225% dry powder, respectively. As the aim of the study was to obtain materials with a defined solid consistency, the maximum amount of hyaluronic gel was selected based on preliminary tests assessing structural changes. The reference samples were, respectively, systems containing only gel (H0%) and emulgel (E0%) without hyaluronan addition. Blue spirulina solutions were added to the samples as a colorant to assist in determining the effect of sample composition on color changes.

Because FUR and HA differ in terms of their thermal stability, their aqueous solutions were prepared separately. Furcellaran solutions were produced by dissolving the polymer in water and mixing on a magnetic stirrer (MR Hei-Tec, Heidolph Instruments GmbH & Co. KG, Schwabach, Bayern, Germany) for 12 h at 80 °C. A 1.5% sodium hyaluronate gel was prepared by dispersing the powder in water and stirring for 1 h at 60 °C. After hydration, all components—at the proportions listed in Table 1—were blended for 5 min at 15,000 rpm using a Polytron PT2500E homogenizer (Dan-Lab, Białystok, Poland). The resulting mixtures were poured into molds and cooled at 5 °C. Following 12 h of cooling, the formed samples were transferred to sealed containers and stored under refrigeration until analysis.

### 2.3. Zeta Potential

The zeta potential of the emulsion and gel solutions prior to cooling was determined using dynamic light scattering (DLS) with a Zetasizer Nano ZS instrument (Malvern Panalytical, Malvern, Worcestershire, UK). Measurements were performed in optically uniform polystyrene cuvettes (measurement range 0.6 nm–6 μm) at a detection angle of 173°. Before analysis, the samples were diluted to a 2% concentration using hot water and mixed on a magnetic stirrer (MR Hei-Tec, Heidolph Instruments GmbH & Co. KG, Schwabach, Bayern) at room temperature until a uniform dispersion was obtained.

### 2.4. Swelling Ratio

The swelling ratio of the emulsion gels and hydrogels was evaluated by immersing a single sample (approximately 10 g) in a 0.02% sodium azide aqueous solution for 48 h. The swelling ratio (SR) was calculated using Equation (1):

(1) $$SR=\frac{w_{2}-w_{1}}{w_{1}}\times 100\%$$

where $w_{1}$ represents the initial weight of the sample and $w_{2}$ denotes the weight after swelling.

### 2.5. Fourier-Transform Infrared (FT-IR) Spectroscopy

The FT-IR spectroscopy of the gels and emulsion gels was determined by using Nicolet IS5 Spectrophotometer (ThermoFisher Scientific, Waltham, MA, USA). The samples were prepared by cutting them into 1 mm slices and drying under a fume cupboard (room temperature, 24 h). The infrared spectra were recorded at a wavelength in the range of 700–4000 cm^−1^.

### 2.6. Scanning Electron Microscopy (SEM)

The morphology of furcellaran–hyaluronic acid gels and emulgels was evaluated by using a Quattro SEM microscope (Thermo Fisher Scientific, Waltham, MA, USA) equipped with FEG (field emission gun) and ColorSEM systems. The hydro- and emulgels were examined in naturally hydrated forms without a spattering of conductive coatings due to the environmental mode (ESEM), where water vapor was injected into the microscope chamber to stabilize the pressure of 200 Pa. The LVD (low-vacuum SE) detector collected SEM cross-sections at magnifications of 75 to 800× with a 20 kV acceleration.

### 2.7. X-Ray Diffraction

X-ray diffraction measurements were carried out using a D8 Advance ECO diffractometer (Bruker, Karlsruhe, Germany) equipped with Cu Kα radiation (λ = 1.54060 Å) and operating in Bragg–Brentano geometry. Wide-angle X-ray diffraction (WAXD) patterns were recorded at ambient temperature over a 2θ range of 5–100°, corresponding to a d-spacing range of 1.01–17.66 Å, calculated using Bragg’s equation as follows:

λ_Cu_ = 2*d* × sinθ (2)

### 2.8. Color Measurement

The surface color of the samples was assessed in five replicates using reflectance mode with a Color i5 spectrophotometer (X-Rite, Grand Rapids, MI, USA; illuminant D65). The CIE-LAB color space was applied to determine the following coordinates:

L*—lightness,

a*—red/green component,

b*—yellow/blue component.

From these values, the hue angle (H°) and chroma (C*) were calculated as follows:

(3) $$H^{\circ }=arctan(\frac{b^{*}}{a^{*}}),C^{*}=\sqrt{(a^{*}{)}^{2}+(b^{*}{)}^{2}}$$

The whiteness index (WI) and yellowness index (YI) were determined using the following equations:

(4) $$WI=\sqrt{(100-{L^{*})}^{2}+(a^{*}{)}^{2}+(b^{*}{)}^{2}}$$

(5) $$YI=\frac{142.86\times b^{*}}{L^{*}}$$

### 2.9. Texture Profile Analysis (TPA)

The textural characteristics of the hydrogels and emulgels were assessed using a two-bite compression test performed on a Shimadzu EZ Test EZ-LX universal testing machine (Shimadzu, Kyoto, Japan) equipped with a 500 N load cell. Each sample was compressed twice to 50% of its initial height using a cylindrical probe with a diameter of 36 mm, ensuring deformation of the gel structure without puncturing it. Texture attributes—including hardness, springiness, cohesiveness, and gumminess—were determined with Trapezium X software (Shimadzu, Japan). All measurements were conducted in quintuplicate.

### 2.10. Large Deformation Mechanical Analysis

Uniaxial compression tests were performed using universal testing machine EZ-Test (Shimadzu, Japan). A cylinder (30 × 30 mm) and a plate were used at a rate of 1 mm/s. Emulgel and hydrogel samples (height 20 mm) were compressed until a 50% deformation rate was achieved while resistance force versus distance were recorded. Based on obtained curves, mechanical charactering parameters were determined as follows: fracture stress (σ), fracture work (W), elastic modulus (E), and fracture displacement. Each sample was measured at five replicates.

### 2.11. Rheological Evaluation

The rheological behavior of the hydrogels and emulgels was investigated by an RS-6000 rotational rheometer (Thermo Fisher, Karlsruhe, Germany) using a parallel plate (1.9 mm gap, 35 mm diameter) geometry probe at 20 °C. Before analysis samples were conditioned at room temperature and cut into slices (height 2 mm and diameter 0.35 mm), the stress sweep tests were performed in oscillation stress sweep mode at frequency of 1 Hz and strain amplitude range from 0.01 to 1.00. The intersection of G′ and G″ curves was recorded as yield stress of the tested materials. The frequency stress was measured with 0.005 applied strain, which was within the linear viscoelastic region. The frequency range was from 0.1 to 100 Hz. All samples were tested in triplicate.

### 2.12. In Vitro Digestion

An in vitro static digestion model was carried out according to Minekus et al. [21]. A 5 g sample was used for digestion. The test was divided into three phases: oral, gastric, and intestinal. The oral phase lasted 2 min, the gastric phase lasted 2 h, and the intestinal phase lasted 2 h. During the experiment, the temperature was maintained at 37 °C. The samples were continuously mixed using a rotary shaker. The obtained digestates were used for further analysis.

### 2.13. Simulation of the Intestinal Absorption Process Using Caco-2 Cells

For the intestinal absorption experiment, a monolayer of Caco-2 cells was used, and after 21 days of culture on inserts, the sample mimics the morphology and physiology of intestinal enterocytes. The digested sample was placed on the top of the Caco-2 cell monolayer. After 2 h, the resulting filtrate was collected. Collected filtrate was then used for mitochondrial membrane potential analysis.

### 2.14. Cell Culture and Treatment

HT-29 human colon adenocarcinoma cells were obtained from the American Type Culture Collection (ATCC, Manassas, VA, USA). The cells were maintained under controlled conditions in the recommended culture medium supplemented with 10% fetal bovine serum (FBS), following ATCC guidelines.

To evaluate the impact of the composite gels on mitochondrial membrane potential, HT-29 cells were seeded at a density of 1 × 10^5^ cells per well in 12-well plates and incubated in growth medium for 24 h. The medium was then replaced with absorbed digestates of the tested samples for a 2 h exposure period. Untreated cells served as the negative control (NC), whereas cells treated with 1.5 µM staurosporine were used as the positive control (S) to induce apoptosis.

### 2.15. Analysis of Mitochondrial Membrane Potential

The effect of composite gels enriched with varying amounts of HA on the mitochondrial membrane potential in HT-29 cells was assessed using the Muse™ MitoPotential Assay (Merck Millipore, Billerica, MA, USA; Catalog No. MCH100110). Measurements were performed with the Muse™ Cell Analyzer (Merck Millipore, Billerica, MA, USA).

Mitochondria are highly sensitive to cellular stress, and alterations in their membrane potential represent an early indicator of apoptosis. The Muse MitoPotential Assay uses a lipophilic cationic dye in combination with 7-Aminoactinomycin D (7-AAD), allowing simultaneous detection of mitochondrial depolarization and loss of membrane integrity, which are two key markers of cell injury and cell death.

### 2.16. DPPH Free Radical Scavenging Capacity Assay

The antioxidant activity of the composite gels with different HA concentrations was evaluated by determining their ability to neutralize the stable free radical 1,1-diphenyl-2-picrylhydrazyl (DPPH) [22]. Extracts of hydrogel or emulgel samples (1 mg/mL) were prepared by dissolving them in deionized water in a hot water bath (50 °C, 30 min). Two milliliters of each extract or digested material were then mixed with 2 mL of DPPH ethanolic solution (100 µmol/L). The mixtures were vortexed for 1 min and incubated for 30 min at room temperature in the dark. Following incubation, samples were centrifuged at 8000× *g* for 10 min, and the absorbance (A) of the supernatant was measured at 515 nm using a Helios Gamma UV-Vis spectrophotometer (Thermo Electron Corp., Cambridge, UK). Control solutions consisted of 2 mL ethanol mixed with 2 mL DPPH solution.

The radical scavenging activity was calculated according to the following equation:

DPPH scavenging activity (%) = ((A control − A sample)/A control) ×100%(6)

### 2.17. Statistical Analysis

All experiments were carried out at least in triplicate and the results were expressed as mean ± standard deviation. Statistica (StatSoft, Inc., Tulsa, OK, USA) software version 13.3. was applied to compare mean values (*p* < 0.05) using one-way ANOVA followed by Tukey’s post hoc test. Data series without normal distribution were transformed according to the Box–Cox method.

## 3. Results and Discussion

### 3.1. Physical Evaluation

**Figure 1:**
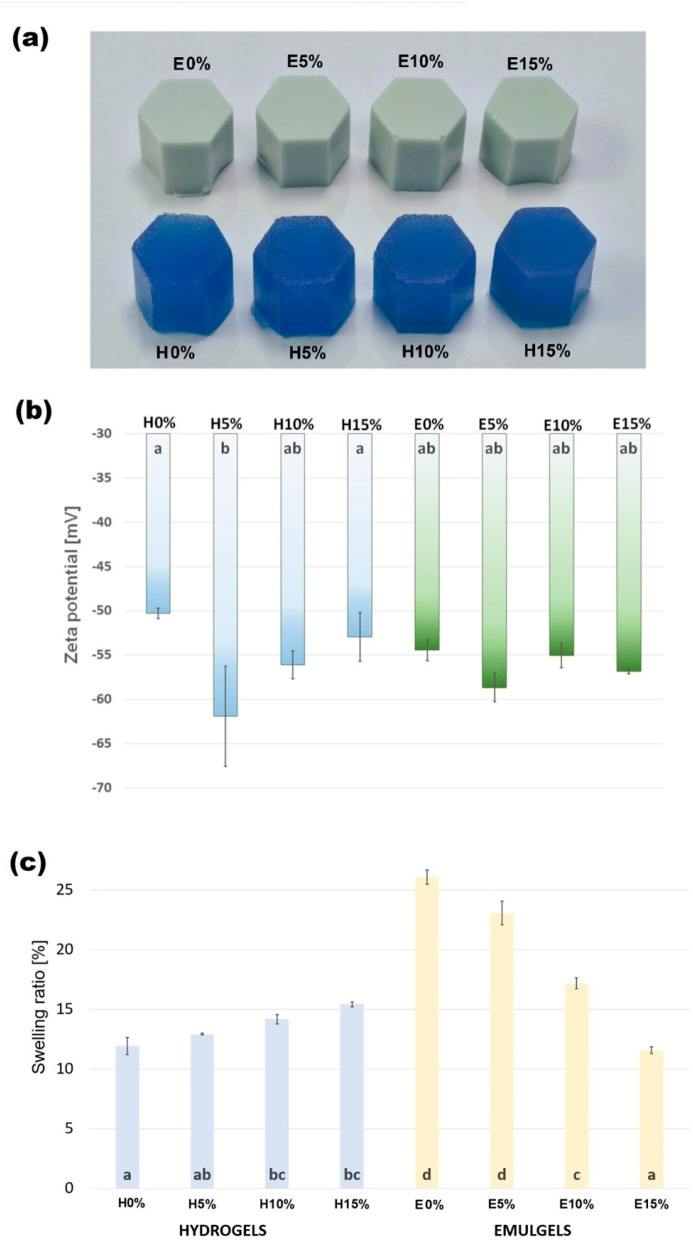
Visual characterization of furcellaran–hyaluronic acid hydrogels and emulsion gels (**a**), zeta potential (**b**), and swelling ratio (**c**). Columns with the same letters are not significantly different (*p* > 0.05).

The visual appearance of spirulina-enriched hydrogels and emulsion gels stabilized by furcellaran with different hyaluronic acid additions are shown in Figure 1a. All used formulations allowed soft but stable structures of the materials to be obtained, with all samples achieving smooth homogenous surfaces and regular shapes. No liquid leakage was observed. The color of the hydrogels was intensely blue and saturated, due to the presence of spirulina. Emulgels containing 10% of evening primrose oil content were a milky-blue shade.

#### 3.1.1. Zeta Potential

The zeta potential parameters for hydro- and emulgels are presented in Figure 1b. Zeta potential is a parameter that reflects the stability of an emulsion. Emulsions with zeta potential values between −20 mV and +20 mV typically have limited stability and are prone to agglomeration. In contrast, emulsions with zeta potential values below −20 mV or above +20 mV are stable and physically more robust [23]. In hydrogels, the addition of HA noticeably increased the absolute value of the zeta potential, with the strongest effect observed at 5% HA. This suggests that HA contributed to improved electrostatic stabilization of the hydrogel-forming solutions.

In emulgels, the incorporation of evening primrose oil shifted the zeta potential toward higher values, indicating an oil-driven modification of interfacial charge. However, in contrast to hydrogels, further addition of HA did not significantly change the zeta potential of the emulsion systems, regardless of the concentration used. This suggests that in emulgels the surface charge is primarily governed by the oil–polysaccharide interface, rather than by HA content.

#### 3.1.2. Swelling Properties

The swelling ratio of biomaterials is related to their internal structure and is an important parameter influencing the kinetics of digestion and the release of bioactive lipophilic substances in the digestive tract. In tissue engineering and biomedicine, it also determines cell amplification and differentiation and, in cosmetic formulations, is reflected in the kinetics of absorption of functional compounds. Values of swelling ratio (SR) obtained for hydro- and emulgels with different levels of hyaluronic acid addition are shown in Figure 1c.

Interestingly, hydrogels and emulgels showed completely opposite characteristics of swelling. Values of SR determined for gels increased with increasing HA addition and varied between 11.92 and 15.41%. The swelling ratios of emulgels were substantially higher and ranged from 26.08%, obtained for samples without hyaluronic acid, to 11.57%, determined for variant E15%. The literature reports on the water absorption properties of HA composite gels are inconclusive and depend on the gelling agent used. Reference [24] proved that the swelling ratio of the sodium alginate-based gels decreases with increasing proportions of HA. In contrast, hydrogels containing bacterial cellulose, like the furcellaran analyzed samples, showed an increase in swelling ratio value with increasing proportions of HA in the material [15]. The increase in SR with increasing additions of hyaluronic acid, which has strong hygroscopic properties, can be simply explained by the increase in available hydrophilic groups in the material. Furthermore, it can be assumed that no bonds were formed between FUR and HA to occupy the hydroxyl groups of the acid. The emulgel reference sample (E0%) showed a swelling ratio that was more than two-times higher compared to hydrogel (H0%). These differences may be due to the fact that, as a result of the addition of oil, the amount of water and thus the amount of hydrophilic groups is automatically reduced, which releases the hydrophilic groups within the polymers of the matrix, allowing them to bind with more of the added water. The presence of oil may also have increased the number of water-retaining micropores formed during homogenization of the gelling solution. The increasing proportion of hyaluronic acid in the material, in turn, resulted in a decrease in the SR values of emulgels. A decrease in the swelling ratio with increasing additions of hyaluronic acid (up to an excess of HA) is also shown for emulsion gels made from whey protein isolate and corn oil. The increasing proportion of HA limiting water retention in the material may be due to the formation of a more compact structure around the fat droplets dispersed in the emulgel matrix, physically blocking access to the hydroxyl groups in hyaluronic acid.

### 3.2. FT-IR Analysis

**Figure 2:**
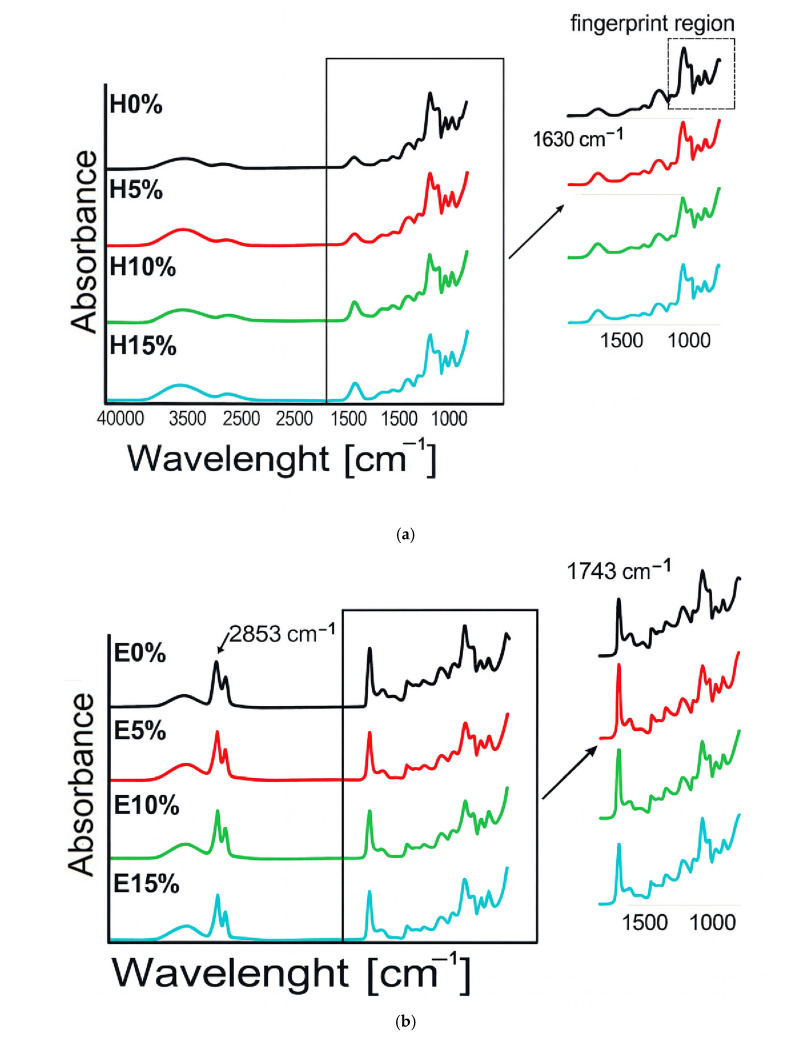

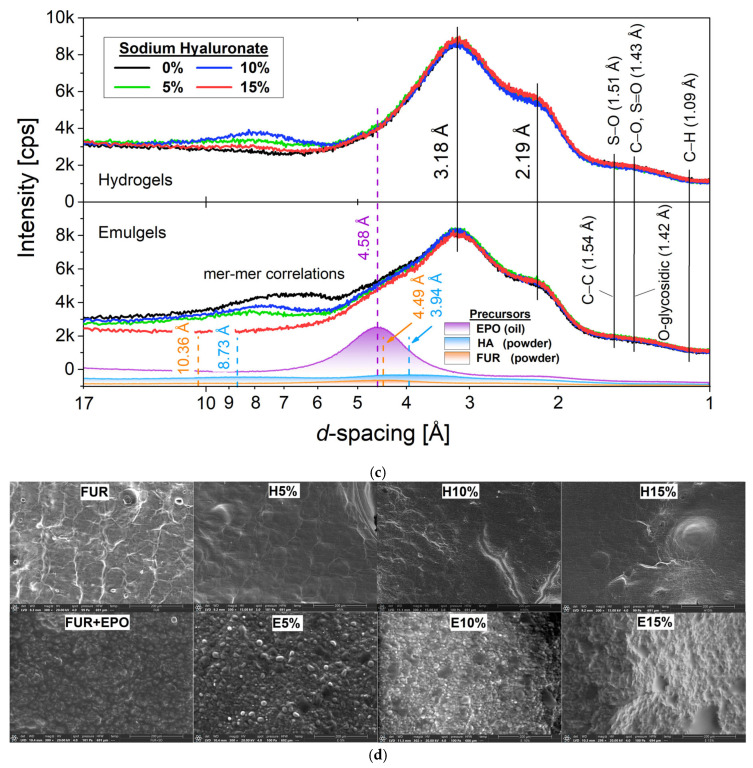
FTIR spectra (**a**,**b**), X-ray diffraction patterns (**c**), and SEM micrographs (**d**) of furcellaran–hyaluronic acid hydrogels and emulsion gels.

The spectrum of furcellaran hydrogels with the addition of hyaluronic acid and emulgels are presented in Figure 2a,b. The spectrum of H0% shows clear peaks: a broad band spreading 3000–3600 cm^−1^ due to a polyhydroxy-OH group, a peak at 2918 cm^−1^ due to C-H stretch, and a peak at 1062 cm^−1^ due to C-O stretch of cyclic ethers, while the peaks at 924 cm^−1^ and 843 cm^−1^ are associated with the stretching mode of SO groups, which is typical of a furcellaran structure [25]. The bands within the 700 to 1000 cm^−1^ range are known as the fingerprinting region, which is distinctive for each polysaccharide [26]. After adding hyaluronic acid to the furcellaran matrix, an increase in the peak intensity at 1630 cm^−1^ was observed, which was attributed to the presence of asymmetrical C=O stretching from HA [27]. The addition of oil influenced the FTIR spectrum of the tested furcellaran materials (Figure 2b). An increase in the intensity of the peaks at 2853 and 1743 cm^−1^ was noted. Additionally, a decrease in the intensity of O-H stretching vibrations was observed in emulgels compared to hydrogels, suggesting an increase in hydrophobic interactions due to the incorporation of the oil phase [28]. The addition of hyaluronic acid to emulgels, only at 5% concentration, significantly increased the intensity of peaks, e.g., at 2800 cm^−1^, 1742 cm^−1^. This behavior suggests that the emulgels with 5% and 10% hyaluronic acid contents were prepared with the optimal composition, which translates into improved mechanical, thermal, and rheological properties of the materials.

### 3.3. X-Ray Diffraction Analysis

Crystalline or amorphous contents could strongly influence water adsorption and swelling characteristics of the biological materials. Therefore, evening primrose oil and hyaluronic acid X-ray diffraction were applied to characterize interaction mechanisms and structural integrity of composite gels containing furcellaran. Moreover, the d-spacing scale (Figure 2c) seems more suitable for characterizing hydrocarbon-base materials due to the organic nature of WAXD patterns without narrow, intense diffracted lines. Hydro- and emulgel ingredients were also investigated by XRD to understand the experimental data reasonably. Pure hyaluronan acid (HA) showed no apparent structural peak for the XRD pattern, indicating its amorphism. Nevertheless, the evident small diffraction line at 8.73 Å that was seen for the HA ingredient, potentially related to the polysaccharide chain organization, was intensified in hydrogel systems. Furthermore, XRD patterns indicate that among hydrophilic samples, the most substantial interactions between HA chains and other components are observed for H10%, while the highest intensity of corresponding Bragg reflections was noticed for emulgels containing no hyaluronic acid. At the same time, the emulsion gel with the highest HA content reveals a flattened XRD pattern, presumably implying stronger hydrogen bonding between biopolymers, consequently resulting in a crystallinity reduction. In turn, pure furcellaran exhibits prominent Bragg reflection at 4.49 Å, demonstrating a more crystalline phase with partial ordering of the polysaccharide chains. The increase in this peak with the HA content, more apparent in hydrogels, suggests polymer matrix rearrangement. In contrast, evening primrose oil exhibits an evident line at 4.58 Å, denoting a β-polymorphic fat fraction type [29]. FTIR and XRD analyses confirmed molecular-level compatibility between furcellaran and hyaluronic acid. The resulting matrices can serve as carriers for both hydrophilic and lipophilic bioactive compounds.

### 3.4. Morphological Properties by ESEM

Morphological characterizations of furcellaran-stabilized gels and emulgels with different hyaluronic acid contents were shown (Figure 2d) using SEM in low-vacuum mode. The ESEM (environmental) mode allows morphology analyses in a natural specimen state without dehydration, which is advantageous for presenting the microstructure of materials with a high cross-linked water content. Discrepancies in microstructure were observed at a magnification of 300 × between emulsion gels and hydrogels, as well as in specimens with different hyaluronic acid concentrations. A hydrogel reference, comprising only furcellaran, spirulina, and water, showed a typical fibrous three-dimensional structure of polysaccharide matrix. Local accumulation of the solids and some spherical pores can also be observed. The progressive incorporation of the hydrogel with hyaluronic acid gradually smoothed the sample surface until the spatial scaffolding of the material composed of polysaccharide chains was no longer visible. Forming a homogeneous surface most likely indicates good compatibility between FUR and HA. However, a few large pores and single deposits of dry substance were observed for the gels with higher HA contents. The porosity of hyaluronic acid gels may be due to aeration of the solution at the homogenization stage. Since furcellaran-based materials were set very quickly at room temperature, removing air bubbles during gel production was impossible. The E0% reference showed rough topography from many irregularly shaped oil droplets trapped in the oil matrix. In contrast, the 5% hyaluronic gel addition made it appear in the microstructure of the emulsion as light-colored near-round particles. Increasing the HA additive to 10% caused its dominance on the gel surfaces while oil-phase droplets were still visible. Interestingly, for E15%, the oil was no longer visible, and gel surfaces consisted only of undulating structures composed of HA particles. Aggregates of hyaluronic acid were also visible, most likely made up of swollen particles, and their entrapment can explain the disappearance of oil droplets in the HA fraction. It is known that hyaluronic acid conjugated with hydrophobic molecules undergoes self-assembling in an aqueous environment to form polymeric micelles composed of hydrophilic coatings (visible in the ESEM image as a light structure) and lipophilic molecules aggregated in the hydrophobic inner core through non-covalent interactions [30].

### 3.5. Color Analysis of Emulgels

Color analysis provides insights into pigment–polymer interactions and stability, which is relevant for food and cosmetic applications. Hyaluronic acid addition effect on the values of hydro- and emulgel color parameters are shown in Table 2. Lightness parameters are considered as an approximate measurement of luminosity, with values varying from 0 for black and 100 for white. The L* values of the composite emulsion gel samples were higher than those of the hydrogels. The increased brightness of gels enriched with vegetable fat is attributed to the light-scattering effect of the emulsion, which is formed when the oil is homogenized with the other ingredients [31]. An increased proportion of hyaluronic acid resulted in intensification of brightness of the emulgel material, while the opposite effect was observed for hydrogels. Hence, it can be assumed that the addition of HA promotes the emulsification process.

The a* parameters of both emulgels and hydrogels showed negative values, which indicates the dominance of green over red hues, and is significantly more intense for samples containing oil. On the other hand, significantly lower b* values, expressing intensity of the blue color, were obtained for variants without the EPO additive. The main component determining the color of the examined systems were the phycocyanins contained in spirulina powder which are responsible for its bright blue appearance. However, differences in the intensity of green and blue tones are caused by the interaction of the pigment with the rest of the components. In emulsion gels, yellow pigments from oil combined with C-phycocyanins intensified the greenness of the material. In hydrogels in which the non-blue pigment was not masked by the presence of oil carotenoids, b* values were significantly higher than the corresponding values determined for emulgels. As the proportion of hyaluronic acid increased, both types of material showed a decrease in the b* value and an increase in the a* parameter. This means that HA has reduced the green color while boosting the blue hue. This trend may be due to the fact that the pH of the material decreases with increasing HA concentration, as a result of which the stability of carotenoid decreased while hydrophilic phycocyanins were stabilized in the FUR-HA complex. The extreme values of hue angle, expressing the difference in a specific color from gray of the same lightness, were determined for control hydrogel (H0%) and emulsion gel (E0%) samples, and in both types of materials, values tended to increase with growing hyaluronic acid concentration. Chroma expressed a measure of whiteness and vividness of color. Values of C* for furcellaran gels ranged from 119.61 to 187.02 and for emulgels from 18.61 to 28.33, indicating that the color intensity perceived by the human eye is several times higher for hydrogels than for samples containing oil. In addition, the effect of increasing the proportion of HA in the material affects the decrease in C* in emulgels while the opposite trend was observed for hydrogel. The white index values of the tested samples showed a similar trend to L* while negative values of yellowness index confirm that none of the examined formulations contained yellow hues, only blue ones.

### 3.6. Texture of Hydrogels and Emulgels

The textural characteristics of semi-solid foods have a significant impact on consumer acceptance, as they allow the sensory experience of consuming the product to be described quantitatively. Furthermore, hardness or elasticity also affects the speed of digestion and the release of nutrients in the digestive tract. Textural parameter values determined for furcellaran hydrogels and emulgels produced with different hyaluronic acid additions are shown in Table 3.

Hardness, as the most important texture parameter, expresses material strength. Its value is calculated as the resistive peak force. For hydrogels stabilized by furcellaran, hardness ranged from 5.92 to 18.77 N, while for emulsions, this value ranged from 4.05 to 11.97 N. In both types of tested systems, it was observed that increasing additions of hyaluronic acid results in decreasing of material strength, since the highest hardness values were determined for samples without HA. Interestingly, incorporating 5% of hyaluronic acid gel into hydrogel resulted in a more than 50% decrease in hardness, while in emulgel, the reduction in this parameter was lower than 5% and showed no significance difference. It has been shown that in protein-containing emulsions, a small addition of HA results in an increase in material strength [14]. In the investigated materials, the opposite tendency was observed. This may be due to the fact that both hyaluronic acid and furcellaran, as anionic polysaccharides, have “competed” in the system for the attachment of water molecules.

However, the highest values of the hardness in sample H5% compared to those of E5% indicates that there is some interaction between HA and oil fraction which influenced gel strength improvement. The interaction between fat and hyaluronic acid in its proton hydrophobic part is used in products for the treatment of dry eye disease and synovial joint lubrication [32]. Springiness expresses the rate of deformation recovery when deformed material goes back to its original form after tension removal. For both hydro- and emulgel systems, values of this parameter increased with increasing hyaluronic acid additions which indicates that HA incorporation has a positive effect on material elasticity. A similar effect of HA was found in emulsion gels prepared with casein [13]. Nevertheless, differences in obtained springiness values were not statistically significant. Cohesiveness expresses resilience of the sample to second deformation during TPA testing and it is considered as an indicator of network integrity and internal bond strength. It can be dependent on ionic influence, hydrogen bonds, as well as hydrophobic interaction in the gel matrix. Cohesiveness may depend on the influence of ions, hydrogen bonds, and also hydrophobic interactions in the gel matrix. Obtained parameter values for furcellaran gels and emulsion gels increased with increasing hyaluronic acid concentration in the material, but higher results were determined for emulgels. This means that the material with the evening primrose oil additions showed more resistance during the second deformation. HA additions to the material strengthened its internal structure, which is a result of the plasticizing effect of fat. Gumminess is a secondary TPA parameter that is proportional to hardness, and was also reflected in the obtained results. The highest values were determined in the samples with the highest hardness levels. Gumminess is a particularly important constant in semi-solid food products, since it indicates the energy required to disintegrate the product during oral consumption. The textural parameters showed that hyaluronic acid additions influenced decreases in structure strength. Hydrogels were harder and more brittle, while emulgels were softer but more elastic.

### 3.7. Large Deformation Properties

**Figure 3:**
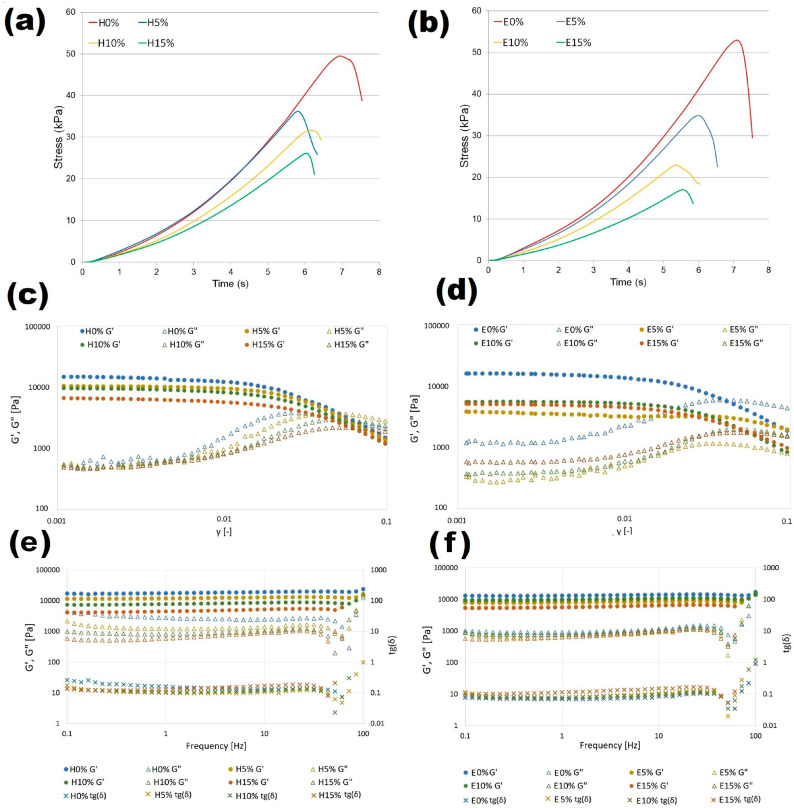
Mechanical and rheological characteristics of furcellaran–hyaluronic acid systems: compression stress–time response of hydrogels (**a**) and emulsion gels (**b**). Stress sweep: hydrogel (**c**) and emulsion gel (**d**) and frequency sweep of hydrogels (**e**) and emulgels (**f**).

The relationship between applied stress and material properties, essential for developing new formulations, designing technological processes, predicting product functionality and stability, can be investigated by the following two approaches. Small strain rheological techniques providing data corresponding to linear viscoelastic regions can be employed to evaluate strength of the intermolecular interactions; however, they do not offer a detailed characterization of the microstructure. Therefore, the development of novel materials for practical application requires knowledge of their mechanical properties specified by large-scale fracture examinations, in order to determine HA influence on the breakdown processes of furcellaran-based hydro- and emulgels. Resistant force–displacement data recorded during uniaxial compression tests converted to the stress–strain curves represent a basis for the determination of mechanical fracture parameters. The stress–time curves determined for hydrogels (A) and emulgels (B) with different hyaluronic acid concentrations are shown in Figure 3. All analyzed formulations show a non-linear course that is characteristic of an elastic material, tending towards the maximum. The course of the curves tends to shift towards the maximum point at which the structure breaks. The effect of increasing HA concentration on both types of gel systems is the same and indicates decreased stress values at time (strain). These results indicate that the addition of HA notably reduced toughness of the composite hydrogels, which may be due to the super hydrophilicity and supramolecular structure of HA macromolecules [33].

Fracture parameters and elastic modulus values of composite gels with different HA concentrations are shown in Table 3. Fracture stress corresponding to the strain at which macroscopic observations of sample rupture were observed, directly express the strength of the material. Fracture work (energy) defines total work of deformation while displacement corresponds to the distance at which the sample cracked. The results from the uniaxial compression test indicate that in both hydrogels and emulsion gels, increasing addition of the proportion of hyaluronic acid had the effect of reducing the strength of the material, which has also been proven for other HA-containing composites [34]. Softening of gels due to increasing proportion of HA may be due to its expanded structure. Values of fracture parameters calculated for samples without hyaluronic acid were slightly higher for hydrogel in comparison to emulsion gel, but the differences showed no statistical significance (*p* > 0.05). However, the decrease in fracture stress and work values as a result of rising HA concentration was more notable in emulgels. The addition of 15% hyaluronic acid to the furcellaran hydrogel reduced the fracture stress value by twofold while a threefold decrease in this parameter was observed for emulsion gel. Elastic modulus values, obtained from the linear range stress–strain curve, decreased with increasing HA addition which indicates enhanced flexibility of the material. Since it was proved that the effect of hyaluronan on biomaterials’ elastic modulus is dependent mainly on its molecular weight [35], it can be assumed that the same addition of HA with a different average molecular weight in the tested systems would totally change their mechanical characteristics.

### 3.8. Rheological Characterization of Emulgels

The results of the strain sweep test used to examine linear and non-linear viscoelastic regions of hydrogels (C) and emulsion gels (D) stabilized by furcellaran with different hyaluronic acid additions is shown in Figure 3. It was observed that at lower strain conditions, storage modulus G′ for all samples was significantly higher than loss modulus, which indicates a solid-like elastic character of the research materials. Based on the large distance between G′ and G″ for the reference samples without HA, it can be concluded that its addition reduces the elastic properties of hydro- and emulgels. Critical linear viscoelastic region (LVR) of all samples was determined at relatively low strain values. After exceeding the LVR point, the elastic G′ of hydrogels and emulsion gels decreased, which indicates that materials yielded or structure damage occurred. A crossover point where values of G′ and G″ were equal indicates that the material showed viscous rheological behavior in the material structure, and this is also referred to as a flow point. The flow point value of tested samples varied around γ equal to 0.05, with one exception. For the emulsion gel prepared with 5% of hyaluronic acid addition, G′ and G″ intersection values were determined at 1.8 (outside the area shown in the figure). Therefore, it can be assumed that the FUR-EPO-HA ratio in the E5% sample provided the most stable material with high self-supporting features. Such properties are desirable for components used in 3D printing filaments.

Figure 3e,f shows the storage and loss modules as a function of frequency obtained for furcellaran-based systems. All curves showed that dynamic moduli were rather independent of the applied frequencies, which is mainly due to the fact that measurements were carried out at room temperature, which is much lower than the gelation temperature. Under such conditions, the structure of the material is stable and affected by the thermal history, hence G′ and G″ reach equilibrium values. In addition, the lack of intersection for the G′ and G″ curves at the studied frequency range indicates that gel to sol transformation has not occurred, which is a typical feature of materials with strong internal forces stabilizing their structure, which is a highly preferable feature with regard to application in food technology. For the entire tested frequency range, every sample exhibited G″ values lower than their corresponding G′ values, confirming their elastic–solid properties. It was shown that an increase in the concentration of hyaluronic acid resulted in a decrease in dynamic moduli values, with the tendency being more pronounced for hydrogels. Thus, it can be assumed that hydrophilic systems with larger volume fractions of uncross-linked native HA domains and the presence of evening primrose oil, attributed to the polysaccharide interaction, improved the stability of the binding gel network. The tight wrapping of the FUR-HA complex around the oil droplets which contributed to their immobilization and prevented flocculation may explain this phenomenon. This result is in line with other composite gel studies [36]. A decrease in viscoelastic properties due to HA addition corresponds with the results of mechanical and textural analyses; however, a loss factor of lower than 1 was obtained for all formulations, indicating that even after applied stress samples were elastic and stable, they did not flow.

### 3.9. Mitochondrial Membrane Potential

**Figure 4:**
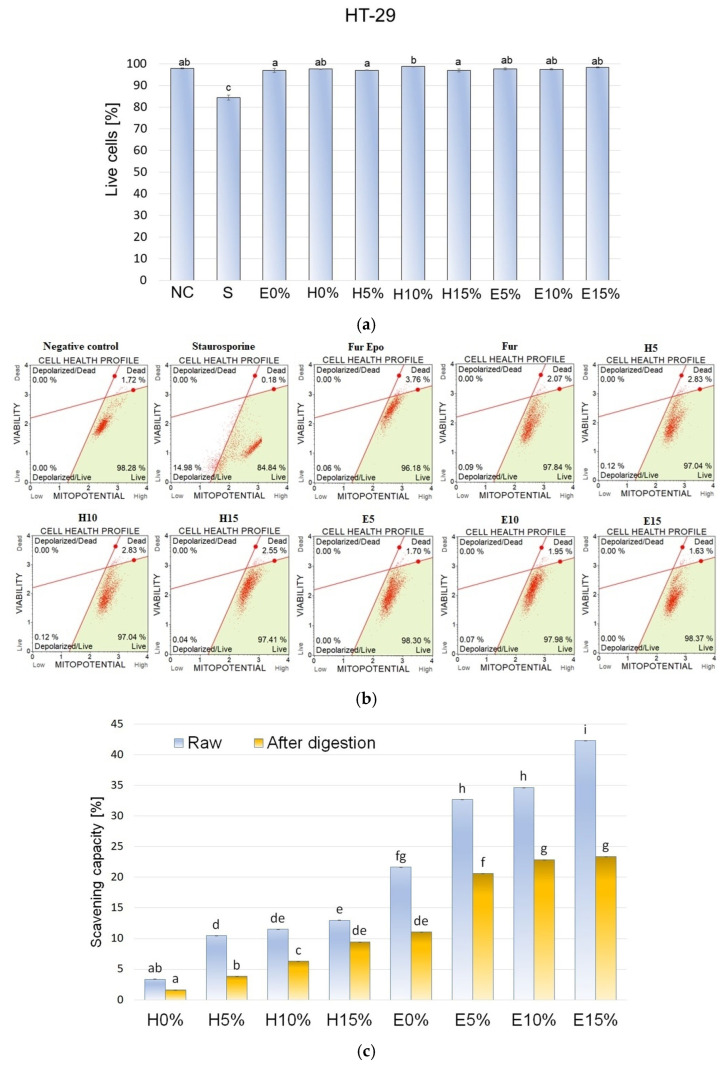
(**a**,**b**) Effect of 2 h exposure to absorbed digestates from different composite gels on mitochondrial membrane potential in HT-29 cells. Data are presented as means ± SD of three independent repetitions. Statistically significant differences were investigated using one-way ANOVA followed by Tukey’s post hoc testing. Columns with the same letters are not significantly different (*p* > 0.05). Staurosporine (1.5 µM) was used as a positive control. (**c**) Antioxidative potential of composite gels with different hyaluronic acid concentration before and after in vitro digestion.

Using the Muse™ MitoPotential Assay (Merck Millipore, Billerica, MA, USA), the mitochondrial membrane potential was measured in HT-29 cells exposed to fraction after absorption on Caco-2 intestinal epithelium. The representative dot plots are shown in Figure 4b. Based on 2 h exposure of cells to absorbed digestates from different furcellaran-based composite gels, it was shown that the mitochondrial membrane potential in HT-29 cells did not change significantly. In comparison to the negative control, no statistically significant changes were noted in the tested cell fractions (Figure 4a). The pool of live cells was maintained at 97-98% and was not significantly different in comparison to the negative control (Figure 4a). Oxidative stress, disruption of mitochondrial respiration, and mitochondrial damage affect aging and cell death in different tissues. Reference [37] demonstrated the protective effects of hyaluronic acid (HA) in primary human chondrocyte cells obtained from osteoarthritis patients that were subjected to oxidative stress induced by 150, 300, and 600 μM peroxynitrite (RNS) and 10, 20, and 40 mU/mL of xanthine oxidase with 0.5 mM hypoxanthine (ROS). Cells pretreated with a medium containing 500 μg/mL hyaluronic acid for 24 h were protected from RNS- and ROS-induced mitochondria DNA (deoxyribonucleic acid) damage in contrast to non-HA-pretreated cells. Similar results were shown for mtDNA repair capacity in HA-pretreated chondrocyte cells exposed to 300 μm peroxynitrite or 20 milliunits of xanthine oxidase and 0.5 mM hypoxanthine. Addition of HA to the culture medium significantly improved the removal of DNA lesions after inducing oxidative stress, which corresponded with increased chondrocyte viability.

### 3.10. DPPH Radical Scavenging Activity

Bioactivity of furcellaran composite gels with different hyaluronic acid concentrations before and after in vitro digestion was evaluated by DPPH assay. As shown in Figure 4c, the antioxidant potential of hydrogel formulation at both tested stages was significantly lower than the bioactivity of the corresponding emulsion gels. Since the initial DPPH scavenging capacity of E0% was almost 85% higher than value obtained for H0%, it can be assumed that those differences were mainly due to the presence of evening primrose oil in emulgels, which exhibit high antioxidant potential [38]. However, increasing concentrations of hyaluronic acid increased the antioxidant potential of both gels and emulsion gels. This phenomenon may be due to the bioactivity of HA itself, as well as its protective effects against fragile compounds that are susceptible to oxidation or light exposure, such as tocopherols found in oils. It can be assumed that the furcellaran–hyaluronic acid matrix, into which the EPO droplets were immobilized, increased its resistance to oxidation by physically isolating the dispersed phase. Apart from EPO, spirulina, used in the formulations as a colorant, also may have been a factor in the antioxidant activity of studied materials. Antioxidant properties of pure HA [39] and its positive effects in complex systems [14] have been reported in other related studies. The DPPH free radical scavenging rate of all gel variants decreased notably after digestion. The smallest reduction of 27% was observed for the H15% variant and the largest reduction of 63% was observed for the H10% sample. The effect of gastrointestinal digestion on bioactivity results from a number of factors, such as material characteristics, pH changes, and interactions with other food components. The obtained results demonstrated that antioxidant activity in the tested composite gels came from digestion-sensitive substances. There was no linear correlation between HA concentration in the sample and the quantitative preservation of biological activity. Oxidative stress resulting from the presence of free radicals can lead to many undesirable changes such as enzyme inactivation, lipids peroxidation, or DNA damage. Therefore, gel complexes with high antioxidant activity are desirable not only in the food sector, but also in the medical (dressings to accelerate wound healing) and cosmetic sector (skin care preparations). The obtained results show the effects of HA on the antioxidant activity of furcellaran-based gels and emulsion gels. Therefore, future research should examine the impact of protein addition and production technology conditions on material bioactivity.

## 4. Conclusions

In this study, six variants of furcellaran-based hydrophilic and amphiphilic composite gels with different hyaluronic acid concentrations were successfully produced. The obtained materials showed stable, solid structures with well-preserved blue hues originating from spirulina. Zeta potential results showed that stability of gel-forming solutions increased as a result of hyaluronan addition. Hydrogel swelling ability increased with HA content while the opposite effect was observed for emulgels, proving that different water-bonding mechanisms occurred in the composite materials. The results of FTIR, XRD, and SEM analyses confirmed homogeneous structure and hydrogen bond stabilization of the hyaluronic acid–furcellaran complex with hydrophobic interactions in emulsion gels. As the HA content in the material increased, the mechanical properties and hardness decreased, while springiness, cohesiveness, and elasticity improved. Small strain rheological results showed that the elastic–solid nature of the samples and emulsion gel formulation containing 0.075% [*w*/*w*] of HA exhibited the highest stability with self-supporting properties. Initial DPPH scavenging activity of the composite gels varied between 3.36 and 42.27% and increased with hyaluronic acid concentration. In vitro digestion resulted in antioxidant activity reduction. Hyaluronic acid incorporation showed no statistically significant effects on mitochondrial membrane potential in HT-29 cells. The results of this study are expected to support the development of novel food-grade materials characterized by a wide spectrum of technological properties determined by formulation. The obtained composite gels can be employed as stable and natural plant-based delivery systems of hydrophilic and lipophilic bioactive ingredients in food, pharmaceutical, and cosmetic applications.

## Figures and Tables

**Table 1:** Furcellaran-based composite gel formulations.

Sample	Furcellaran Content [g/100 mL]	Sodium Hyaluronate 1.5% Gel (g/100 mL)	Evening PrimRose Oil Content [ml/100 mL]	Spirulina [g/100 mL]
H0%	3.0	0.0	0	0.6
H5%	3.0	5.0	0	0.6
H10%	3.0	10.0	0	0.6
H15%	3.0	15.0	0	0.6
E0%	3.0	0.0	10	0.6
E5%	3.0	5.0	10	0.6
E10%	3.0	10.0	10	0.6
E15%	3.0	15.0	10	0.6

**Table 2:** Influence of different hyaluronic acid contents on color parameter values of composite gels.

	L*	a*	b*	H°	C*	WI	YI
H0%	26.18 ± 0.97 ^a^	−1.4 ± 0.11 ^a^	−15.39 ± 0.75 ^a^	84.79 ± 0.56 ^a^	119.61 ± 11.67 ^a^	24.58 ± 1.06 ^a^	−84.15 ± 6.51 ^a^
H5%	23.41 ± 0.58 ^b^	−1.8 ± 0.23 ^b^	−18.01 ± 0.34 ^b^	84.29 ± 0.79 ^a^	163.94 ± 5.95 ^b^	21.3 ± 0.51 ^b^	−109.95 ± 1.68 ^b^
H10%	19.94 ± 0.34 ^c^	−0.61 ± 0.07 ^c^	−18.55 ± 0.79 ^bc^	88.10 ± 0.20 ^b^	172.57 ± 14.56 ^bc^	17.81 ± 0.24 ^c^	−132.91 ± 4.39 ^c^
H15%	18.65 ± 0.15 ^d^	−0.23 ± 0.05 ^d^	−19.34 ± 0.19 ^c^	89.33 ± 0.16 ^c^	187.02 ± 3.60 ^c^	16.38 ± 0.15 ^d^	−148.15 ± 1.93 ^d^
E0%	72.25 ± 0.10 ^e^	−7.51 ± 0.05 ^e^	−0.45 ± 0.02 ^d^	3.44 ± 0.17 ^d^	28.33 ± 0.40 ^d^	71.24 ± 0.09 ^e^	−0.89 ± 0.04 ^e^
E5%	74.89 ± 2.07 ^f^	−6.51 ± 0.19 ^f^	−0.86 ± 0.08 ^d^	7.54 ± 0.75 ^e^	21.55 ± 1.20 ^d^	75.65 ± 0.09 ^f^	−1.60 ± 0.14 ^e^
E10%	77.23 ± 0.48 ^f^	−6.21 ± 0.05 ^g^	−1.85 ± 0.03 ^e^	16.62 ± 0.18 ^f^	21.00 ± 0.36 ^d^	76.32 ± 0.47 ^f^	−3.43 ± 0.07 ^e^
E15%	77.24 ± 0.15 ^f^	−5.62 ± 0.05 ^h^	−2.36 ± 0.04 ^e^	22.8 ± 0.23 ^g^	18.61 ± 0.32 ^d^	76.44 ± 0.13 ^f^	−4.37 ± 0.06 ^e^

Average values ± standard deviation from replicates. Different letters indicate significant differences in the same column (*p* < 0.05).

**Table 3:** Mechanical and textural parameter values of composite emulsion gels.

	Texture			
	Hardness [N]	Springiness	Cohesiveness	Gumminess [N]
H0%	18.77 ± 1.16 ^a^	0.850 ± 0.079 ^a^	0.045 ± 0.013 ^a^	0.868 ± 0.063 ^a^
H5%	9.01 ± 0.81 ^b^	0.922 ± 0.067 ^abc^	0.047 ± 0.002 ^ab^	0.587 ± 0.098 ^b^
H10%	8.12 ± 0.76 ^bc^	0.938 ± 0.039 ^abc^	0.059 ± 0.005 ^abc^	0.545 ± 0.065 ^b^
H15%	5.92 ± 0.56 ^d^	0.980 ± 0.068 ^abc^	0.087 ± 0.007 ^de^	0.254 ± 0.019 ^c^
E0%	11.97 ± 0.41 ^e^	0.902 ± 0.073 ^ab^	0.070 ± 0.015 ^abcd^	0.823 ± 0.093 ^ad^
E5%	11.59 ± 0.86 ^e^	0.933 ± 0.075 ^abc^	0.072 ± 0.008 ^bcd^	0.721 ± 0.015 ^d^
E10%	6.80 ± 0.87 ^dc^	1.056 ± 0.195 ^bc^	0.080 ± 0.004 ^cd^	0.460 ± 0.057 ^be^
E15%	4.05 ± 0.25 ^f^	1.120 ± 0.115 ^c^	0.110 ± 0.020 ^e^	0.362 ± 0.045 ^ce^
	**Fracture** **M** **echanic**			
	**Fracture Stress [kPa]**	**Fracture Work [mJ]**	**Elastic** **M** **odulus [kPa]**	**Fracture** **D** **isplacement [mm]**
H0%	53.20 ± 4.65 ^a^	53.95 ± 5.78 ^a^	76.62 ± 3.43 ^a^	7.24 ± 0.30 ^a^
H5%	37.72 ± 2.87 ^b^	32.73 ± 1.66 ^b^	76.46 ± 4.26 ^a^	6.00 ± 0.24 ^bc^
H10%	33.73 ± 3.27 ^b^	30.85 ± 3.25 ^b^	63.50 ± 1.33 ^b^	6.36 ± 0.29 ^c^
H15%	26.34 ± 0.40 ^c^	23.68 ± 1.31 ^c^	57.71 ± 1.42 ^c^	6.12 ± 0.19 ^bc^
E0%	53.34 ± 1.54 ^a^	53.74 ± 1.11 ^a^	81.76 ± 1.96 ^d^	7.16 ± 0.08 ^a^
E5%	35.59 ± 2.56 ^b^	32.60 ± 3.95 ^b^	73.11 ± 2.15 ^a^	6.16 ± 0.24 ^bc^
E10%	25.90 ± 2.43 ^c^	22.47 ± 3.61 ^c^	60.66 ± 0.84 ^cb^	5.84 ± 0.42 ^bc^
E15%	17.33 ± 1.25 ^d^	14.90 ± 1.14 ^d^	47.65 ± 2.54 ^e^	5.70 ± 0.13 ^b^

Average values ± standard deviation from replicates. Different letters indicate significant differences in the same column (*p* < 0.05).

## Data Availability

The original contributions presented in this study are included in the article. Further inquiries can be directed to the corresponding author.
